# Using systems science methods to enhance the work of national and local walking partnerships: practical insights from Ireland

**DOI:** 10.1093/eurpub/ckac076

**Published:** 2022-08-26

**Authors:** Dylan D Power, Barry M Lambe, Niamh M Murphy

**Affiliations:** Centre for Health Behaviour Research, Department of Sport and Exercise Science, South East Technological University, Ireland; Centre for Health Behaviour Research, Department of Sport and Exercise Science, South East Technological University, Ireland; Centre for Health Behaviour Research, Department of Sport and Exercise Science, South East Technological University, Ireland

## Abstract

**Background:**

Physical activity (PA) literature is dominated by individual-level descriptive studies, which are known to have limited impact on population PA levels. Leveraging systems science methods offers opportunities to approach PA in a manner which embraces its inherent complexity. This study describes how participatory systems mapping and social network analysis (SNA) were used to understand the work of local and national level walking systems in Ireland.

**Methods:**

Two adapted participatory action research workshops with multisectoral stakeholders were used to develop a systems map for walking in Cork, Ireland. The Global Action Plan for Physical Activity 2018–2030 (GAPPA) map was used as a framework to categorize workshop outcomes. Secondly, SNA methods were used to analyse the communication network between partners of Get Ireland Walking, a national walking promotion initiative, as defined within their strategic plan and the actual communication network as experienced by the partners.

**Results:**

The systems mapping process allowed stakeholders to identify 19 suggested actions for the Cork walking system. The SNA found that there were considerably fewer communication ties between partners in the actual communication network than in the strategy defined network.

**Conclusion:**

The systems mapping process was a useful catalyst for engaging stakeholders in cross-sectoral communication and the GAPPA was a practical way to organize workshop outcomes. Social network analysis methods highlighted that the communication network of a national level walking promotion partnership is not working as planned. Overall, the use of systems science methods can provide practical insights for local and national level walking systems.

## Introduction

Global physical activity (PA) levels remain low and have stagnated over the last number of decades.^1^ Although increases in PA research funding have been recorded in some parts of the world, research outputs remain dominated by individual-level descriptive studies which provide little impact for population levels of PA.^2^^,^^3^ Recently, approaches to PA and other public health problems which embraces the inherent complexity of these problems have been called for.^4^ For example, the publication of the Global Action Plan for Physical Activity 2018–2030 (GAPPA)^5^ by the World Health Organization provides practical guidance to understand the multiple influences and intervention points for national PA systems. This policy framework has been used to guide practice in Ireland where Murphy *et al*.^6^ used the GAPPA framework to form the basis of a national effort to organize and coordinate action amongst stakeholders in the PA system. Globally, there is an increase in publications exploring the application of systems thinking to public health and many have advocated for systems approaches to public health problems such as PA.^7^^,^^8^ Systems approaches place emphasis on cross-sectoral collaboration^8^ and require diverse approaches to synthesizing evidence from a range disciplines and study designs.^9^

Framing PA, or specific forms of PA such as walking, from a systems perspective acknowledges that the behaviour is the result of the complex interplay between individual, socio-political, environmental, societal and biological factors.^8^ Methods such as social network analysis (SNA) and participatory systems mapping offer an opportunity to explore and understand the systems which public health problems are embedded. Systems maps are visual representations of a system which are developed by engaging an interdisciplinary group of stakeholders who work within that system.^8^^,^^10^^,^^11^ Social network analysis is a suite of methods that has been used as a way of investigating how interorganizational networks in public health work, by analysing measures such as degree centrality (the number of ties each stakeholder has), centralization (the extent to which the network is centralized around few organizations) and network density (the overall degree of interconnectedness of the network).^12–15^

There is a paucity of literature highlighting the practical utility of tools such as systems maps and SNA applied to PA. Cavill *et al*.^10^ developed a systems map with local level PA stakeholders in the UK to identify actionable outcomes for the system. This systems map focused on elucidating the direction of the relationships between factors, facilitating a more in depth understanding of the inherent behaviour of the system. Although this process proved useful, little is known about the extent to which existing systems maps can be applied to other contexts, which may act as a useful starting point for researchers and practitioners in the field who may be uncertain regarding the application of systems methods.^16^ Furthermore, a recent systematic review concluded that SNA not only provides benefits for researchers interested in PA but also for practitioners involved in the promotion of PA.^14^ While there are many barriers to effective multidisciplinary partnerships in public health,^17^ SNA methods can allow researchers to gain insight into who the gatekeepers of resources and information within PA promotion networks are.

Enhancing and increasing walking is important across several sectors and its promotion is not the job of any one agency or system. Similar to PA,^11^ walking is associated with transport and for human movement; for sport and recreation; for community-wide initiatives; and tourism, liveability and urban design. To this end, understanding walking promotion from a systems-based perspective may hold benefits which transcend physical and mental health.^18^^,^^19^ Thus, the nature of walking-related work in Ireland is decentralized, meaning that no single organization is responsible for all walking-related programmes, infrastructure or events and it may be seen as the concern of many yet the responsibility of none. The Irish Sports Council (now Sport Ireland) was established in 1999^20^ with a remit for both sport and PA.^21^^,^^22^ In 2013, a national walking promotion organization, Get Ireland Walking (GIW), was established within the national governing body for Irish hillwalking and mountaineering, Mountaineering Ireland, with the aim of coordinating the work of intersectoral organizations with a direct and indirect role in walking in Ireland. However, the national and local structures are fragmented and the dynamics of how these partnerships work is unknown.

The current work aims to utilize methods from systems science to facilitate a holistic understanding of the nature of walking promotion in Ireland. Specifically, this article describes how two methods, participatory systems mapping and SNA, were used to understand the work of national and local walking systems in Ireland.

## Methods

SNA methods were used to analyse the structure of the network between partners and collaborators in Get Ireland Walking’s Strategy and Action Plan 2017–2020 (SAP),^23^ compared with the actual communication network as experienced by the partners. An adapted participatory action research (PAR) methodology was used to develop a systems map for walking in County Cork, Ireland. Ethical approval was granted by the School of Health Sciences Ethics Committee at South East Technological University, Ireland.

### Systems map development

An adapted PAR methodology using two participatory online workshops modelled from previous work^6^ was used to develop a systems map for walking in County Cork, Ireland. Participatory action research is a useful way of exploring problems within public health due to the involvement of stakeholders in co-designing solutions.^24^

#### Population and sampling

Cork (population approximately 540 000) is the largest county in Ireland and was the geographical boundary for which the systems map was developed.^25^ Cork is located on the south-west coast of Ireland and contains a city, multiple largely populated towns, mountain ranges and coastal areas. A local walking promotion officer assisted in purposively recruiting multidisciplinary stakeholders whose role was associated with walking, either directly or indirectly (*n* = 32) to attend the systems mapping workshops. Therefore, walking was broadly defined to include recreational and transport walking to ensure the inclusion of stakeholders from multiple sectors. The specific areas of work for all workshop participants are outlined in Supplementary file S1.

#### Procedures

The process was guided by applying a pre-existing systems map for PA^11^ to the Cork context. The Australian Systems Approaches to Physical Activity Systems Map (ASAPa)^11^ outlines a range of factors which influence PA ranging from individual level factors (demographic status, physiology and psychology) to systems level factors (political environment and governance, transparency and accountability) and the complex network of interconnections between them. The ASAPa Systems Map for PA outlines eight system intervention points which are areas within the PA system where interventions can be implemented. The eight system intervention points are (i) Transport and Human Movement Environment, (ii) Workplaces, (iii) Community-wide Programmes, (iv) Education, (v) Sport and Recreation, (vi) Primary and Secondary Healthcare, (vii) Mass Communication and Public Education and (viii) Physical Environment, Urban Design, Liveability and Walkability.^11^

Two participatory online workshops were facilitated using the Zoom video-conferencing^26^ platform. The central question posed to attendees of workshop one was ‘What interventions are currently being implemented successfully in Cork to promote walking?’. Workshop one lasted 75 min and involved open discussion between stakeholders (*n* = 5) focusing on examples of good practice which existed in each of the eight system intervention points of the ASAPa Systems Map for PA.^11^ The main purpose of workshop one was to develop the first iteration of the map which was designed by the lead researcher using the Kumu.io^27^ software package following the collation of identified interventions. Any duplicate or conflicting suggestions were discussed and a consensus was reached by the authors before the systems map was circulated to all participants who attended workshop one for approval. Participants could access the interactive map via web-link and adjust the map prior to the second workshop.

The central question posed to stakeholders (*n* = 16) in the second workshop was ‘What should be done to help increase overall walking levels going forward in Cork?’. Breakout rooms were labelled by combining the eight system intervention points from the ASAPa Systems Map for PA with participants being allocated to each breakout room according to their expertise. The breakout rooms were; (i) Recreation, Community Wide Programmes and Mass Communication and Public Education, (ii) Primary and Secondary Healthcare, Education and Workplaces and (iii) Physical Environment, Urban Design and Liveability, and Transport and Human Movement Environment. One facilitator was assigned per breakout room who facilitated discussion and took notes. Workshop two lasted 120 min. Following the second workshop, a meeting was convened between the lead researcher and the facilitators of the breakout rooms to develop a second and final iteration of the systems map using the Kumu.io^27^ software, which was circulated to all workshop participants for amendment and approval.

#### Data analysis

Thematic analysis (TA) was used to analyse workshop outcomes due to its highly flexible nature which can be modified for the needs of a particular study.^28^ The analysis of data resulting from the workshops followed the process undertaken by Murphy *et al*.^6^ who used a deductive TA approach to assign outcomes from a national PA systems workshop in Ireland to the areas of the GAPPA.^5^ The GAPPA^5^ is a framework for action which outlines 20 multidimensional policy actions which are encompassed within four strategic objectives (Create Active Societies; Create Active Environments; Create Active People; and Create Active Systems) which capture a whole-of-systems approach to increasing PA. The categorization of examples of good practice and suggested actions identified by stakeholders during workshop two was predetermined by the quadrants of the GAPPA systems map for PA and specific actions outlined within the GAPPA.^5^ Consensus was reached on the appropriate quadrants and actions within the GAPPA framework by all authors.

### Social network analysis

Social network analysis is a suite of tools used to understand the dynamics of various networks, ranging from biological to human social networks.^12^ Social network analysis methods were used to compare the strategy defined network between organizations as written within GIW’s SAP (desktop exercise) and the actual communication network as experienced by the organizations (survey).

#### Population and sampling

All organizations listed in the GIW SAP (*n* = 30) were included in the strategy defined network. Partner organizations of GIW in 2021 (*n* = 33) were purposively recruited to take part in a partnership evaluation survey in March 2021.

#### Procedures

To develop a network diagram for the communication network as written in the SAP, organizations listed as partners/collaborators on the same action were assumed to have communication ties between them. To collect network data for the actual communication network between partners, a partnership evaluation questionnaire (adapted from a pre-existing public health partnership evaluation tool^29^) was sent to 33 participants (*n* = 19 responses, 70% response rate). Respondents were provided with a list of all organizations within the GIW network and were required to list up to 10 organizations they had communicated with in the last 6 months in relation to the GIW SAP*.* Adjacency matrices were developed from both networks and imported into R.^30^

#### Data analysis

Network density, degree centrality and centralization were calculated for both networks using the package ‘igraph’ in R.^30^ The Fruchterman–Reingold layout was used for the network diagrams which places the nodes with the highest centrality scores in central positions.^31^

## Results

### Systems mapping

Stakeholders identified 39 ‘existing examples of good practice’ interventions in the Cork walking system in workshop one. A total of 19 suggested actions were identified as opportunities to improve the system of walking in Cork in workshop two.

**Figure 1:**
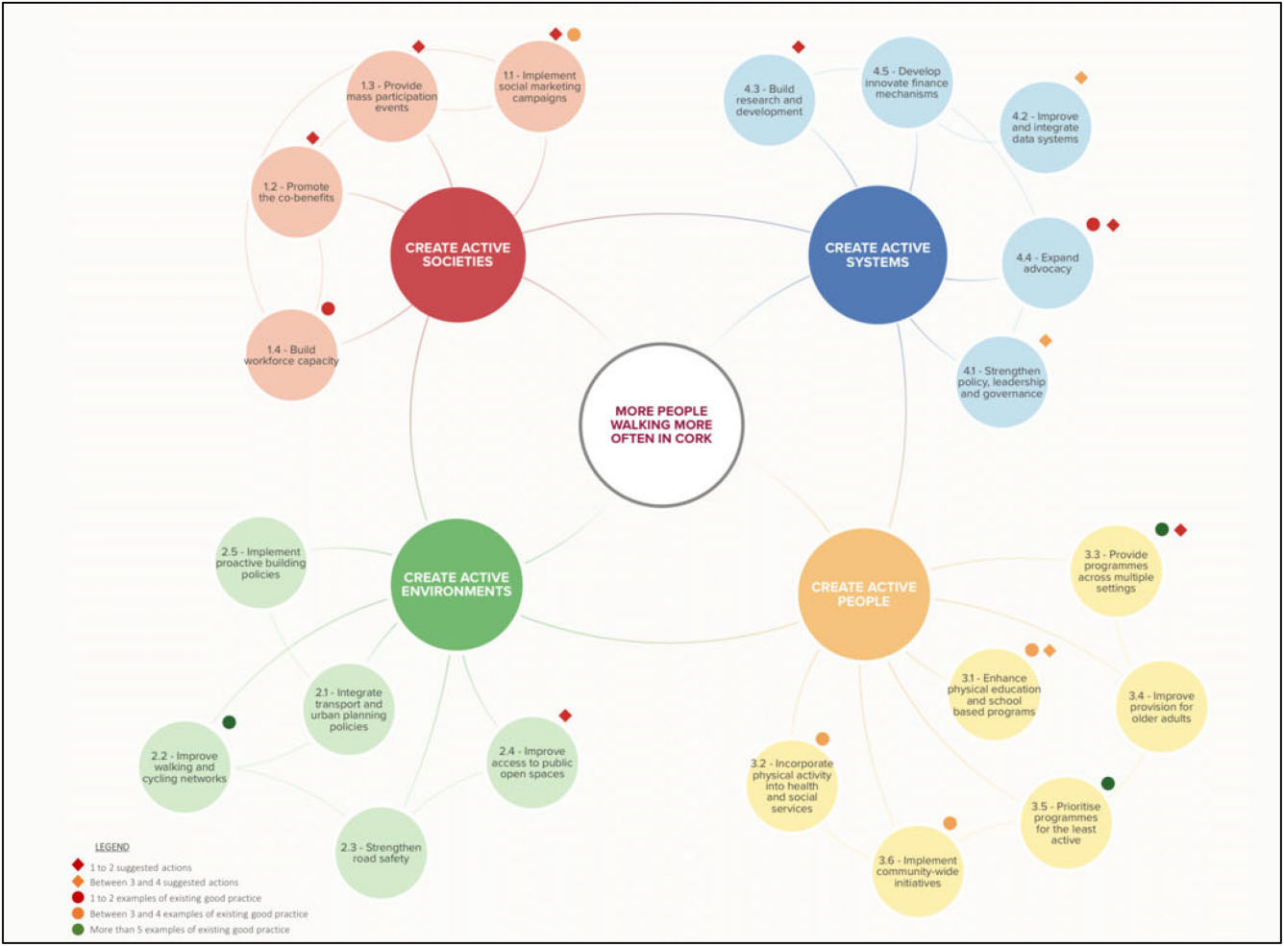
Examples of good practice and areas of suggested action plotted on the GAPPA systems map for PA (World Health Organisation, 2018)

The outcomes of workshop one (examples of existing good practice) and workshop two (suggested actions) are presented according to the quadrant and specific action of the GAPPA^5^ for which it may have the most impact (figure 1). Most examples (56%) of existing good practice within the Cork walking system were individual level programmes (Create Active People). For example, multiple community-based walking programmes were highlighted as examples of good practice by many stakeholders. The majority (58%) of the suggested actions identified by the stakeholders were relevant to the Create Active Systems quadrant of the GAPPA systems map for PA. Twenty-one percent and 16% of suggested actions fell within the Create Active People and Create Active Societies quadrants, respectively. One (5%) solution fell within the Active Environment quadrant of the GAPPA systems map for PA. Examples of suggested actions include regular meetings between local government representatives and stakeholders, and integrating a standard evaluation framework throughout the evaluation of interventions in Cork.

### Social network analysis

**Figure 2:**
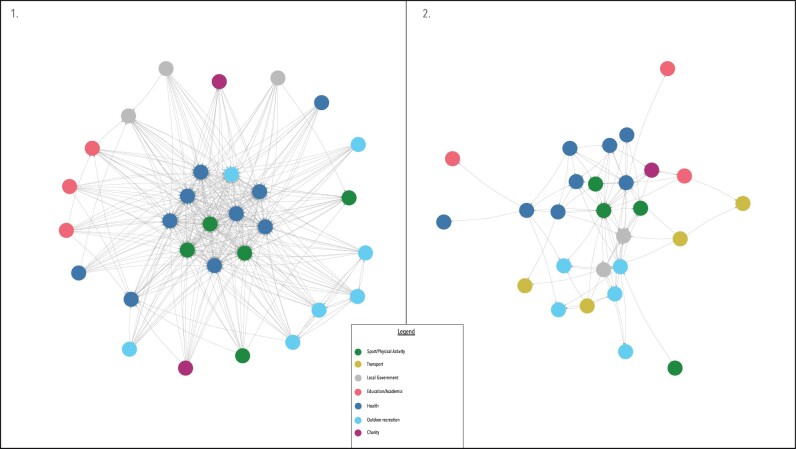
Network diagrams of (Plot 1) strategy defined communication network and (Plot 2) actual communication network experienced by the partners

Figure 2 represents both network diagrams for the communication network between partners as written in the SAP (Plot 1; figure 2) and the actual communication network as experienced by the partners (Plot 2; figure 2). There were considerably fewer communication ties in the actual network than in the strategy defined network. The network density score for the strategy defined network was 0.41, representing a moderate to high-density score.^32^ The network density score for the actual communication network partners was 0.13, which is considered a low level of density.^32^ Both networks also differed on how centralized they were around few organizations. Degree centralization scores were 26.92% (actual communication network) and 40.92% (written communication network). These scores indicate that the strategy defined network is moderately centralized around a group of 11 organizations, whereas the actual communication network had a lower centralization score around four organizations. The 11 organizations who were central to the strategy defined communication network were from the Sport/PA, Health and Outdoor Recreation sectors. However, the actual communication network indicated that organizations from Local Government were among the central organizations in the network, contrary to the strategy defined network.

## Discussion

This article illustrates how systems science methods were used to understand local and national walking systems in Ireland. Firstly, the systems mapping process highlighted that the majority of good practice examples of interventions within the Cork walking system lie within the Create Active People (individual level) quadrant of the GAPPA framework. The systems mapping process also allowed stakeholders to identify suggested actions for their system, more than half of which were directed at the Create Active Systems quadrant. Secondly, the SNA highlighted that there were fewer communication ties in the actual communication network compared with the strategy defined network (network density) and that the communication was centralized around fewer organizations than intended (degree centralization).

Many systems maps have been developed to understand local and national PA systems.^8^^,^^10^^,^^11^ The systems mapping process presented here was a useful way of allowing stakeholders to situate themselves and their work within the system and more importantly, to identify tangible solutions and actions to address barriers within their system. The ability for existing systems maps, such as the ASAPa systems map for PA^11^ and the GAPPA systems map for PA,^5^ to be applied to other contexts and provide a platform upon which to base context-specific discussions is a valuable learning from this process. However, it must be highlighted that the workshop outcomes are a result of discussions between stakeholders who were present in the workshops, and a different group of stakeholders may produce different outcomes. For example, the lack of representation from stakeholders from the areas of Transport and Human Movement, and Physical Environment, Urban Design and Liveability, may explain the few suggested actions in the Create Active Environments quadrant of the GAPPA framework.^5^ Murphy *et al*.’s study^6^ which describes the process of a systems approach to increase PA in Ireland is one example of using the GAPPA^5^ as a framework for their analysis. GAPPA^5^ provides a mechanism for organizing the outcomes of systems mapping processes and may help to provide consistency across the expanding literature base investigating the application of systems science methods to public health problems. While employing a deductive TA approach may give a superficial description of data,^28^ the GAPPA^5^ proved beneficial in providing a structure to guide the analysis. The challenge remains of tracking the overall implementation of a systems approach.^16^^,^^33^ During the months following the workshops, a steering committee of 10 representatives from multidisciplinary organizations was created and chaired by a part time walking promotion officer in Cork. This steering group continues to monitor the implementation of identified actions by collecting data such as stakeholder engagement in meetings, meeting minutes, and action delivery monitoring. These results will indicate whether the systems approach was, indeed, effective in solidifying collaborative action.

The majority (58%) of the suggested actions identified by stakeholders within the walking system in Cork were situated within the Create Active Systems quadrant of the GAPPA.^5^ Although stakeholders acknowledge that governance, political structures and the knowledge environment require improvement, these types of interventions have been noted as the most difficult to implement.^34^^,^^35^ Changes to the higher-level goals of systems (including stakeholders’ worldviews) require sustained and adaptive multidisciplinary efforts over multiple political cycles for systems change to occur, which is not consistent with the short-term requirements of funding agencies. Furthermore, inherent to a systems approach to PA is an acknowledgement that all factors within a system are interconnected and no one policy solution to reduce physical inactivity exists.^5^ The suggested actions offered by the stakeholders from the systems mapping workshop within the Create Active Systems quadrant may impact—and are interdependent—with the activities within the Create Active People quadrant. However, the lack of engagement with higher-level goals is evident in the current findings. For example, the majority of the examples of good practice interventions identified within the Cork walking system were individual level programmes and interventions, which are known to produce modest population level behaviour change.^2^ The current study outlines the process by which a pre-existing systems map was applied to a novel context without placing emphasis on understanding the directionality between nodes of a systems map. Although there are benefits to increasing the specificity of the systems map to gain a deeper insight into the behaviour and inherent dynamics of the system,^36^ the technicalities of engaging with unfamiliar methods have been noted to be a potential deterrent for stakeholders in local and national public health systems to adopt such methods.^16^

The systems mapping process allows stakeholders to get the ‘lay of the land’ and to allow communication networks across sectors to grow. However, what the systems mapping process does not allow for is an understanding of how stakeholders collaborate and communicate across a system. Social network analysis methods have been used to address this by understanding who the central and peripheral organizations are within PA, healthy living and obesity prevention networks.^13–15^ Similar to the work presented here, the work of Loitz *et al*.^15^ found low-density scores in funding and partnership networks in a group of multidisciplinary stakeholders promoting active living in Alberta, Canada. Using SNA methods in the manner presented in this article may act as a useful way of assessing the extent to which partnerships are working as planned. For example, one network presented in this article represents all communication ties between organizations due to collaborate on actions together as defined within a national walking promotion strategy. Our results show that there is a mismatch between the strategy-defined communication network and the network experienced by the partners within it. However, it must be noted that missing data may skew network density scores.^12^ In the strategy-defined network, there were 11 core organizations that make up part of the central group of organizations, whereas in the actual network, 4 organizations were found to represent the focal point of the network. Furthermore, local government organizations were noted as key players in the actual communication network, yet these are not well represented in the strategy defined network. Such insight allows partnerships to address this inconsistency by developing mechanisms to improve the diffusion of information and facilitating communication across the network by targeting organizations who are most central.^37^^,^^38^

The purpose of this article was to illustrate how systems mapping and SNA were used to understand the work of local and national partnerships for walking in Ireland. This study highlights the utility of using the systems mapping process to engage local level stakeholders, to identify suggested actions to improve the system and a structure for monitoring these actions. The practical insights gained from the SNA process are 2-fold. Firstly, using SNA to understand the dynamics of strategy defined communication networks provides another way of monitoring policy implementation. Secondly, SNA can identify key players in PA and public health organizational networks. Overall, this article provides a real-world example of the application of methods from systems science to understand national and local walking systems in Ireland.

## Supplementary data

Supplementary data are available at *EURPUB* online.

## Funding

Dylan Power is a recipient of a PhD scholarship which is co-funded by Get Ireland Walking and South East Technological University.

*Conflicts of interest*: None declared.

## Data availability

The data used in this article will be shared upon reasonable request to the corresponding author.

Key points

- The process of building a systems map acts as a catalyst for cross-sectoral communication and helps identify mutually beneficial actions with multidisciplinary stakeholders in a local level walking system.
- The utilization of existing systems maps can both accelerate the systems mapping process and ensure that the identified action points cover all levels identified in the Global Action Plan for Physical Activity 2018–2030 (GAPPA).
- Public health partnerships can use social network analysis methods to monitor the implementation of their work by identifying strengths and weaknesses in their communication networks.

## Supplementary Material

ckac076_Supplementary_Data
