# Supplementary material for: Using systems science methods to enhance the work of national and local walking partnerships: practical insights from Ireland
Source: Eur J Public Health. 2022 Aug 26;32(Suppl 1):i8–i13. doi: 10.1093/eurpub/ckac076 (PMC9421407; doi:10.1093/eurpub/ckac076)
Supplement: ckac076_Supplementary_Data [file ckac076_supplementary_data.docx]

Table 1 - List of participants at workshop 1 and workshop 2. WS1 = Workshop 1, WS2 = Workshop 2

| # | **Role** | **WS1** | **WS2** | **Main area of work** |
| --- | --- | --- | --- | --- |
| 1 | Walking Promotion Officer | X | X | Sport and Recreation |
| 2 | Health Promotion Officer | X | X | Primary and Secondary Healthcare |
| 3 | Programmes Manager | X | X | Sport and Recreation |
| 4 | National Programme Manager | X | X | Sport and Recreation |
| 5 | Sports consultant | X | X | Sport and Recreation |
| 6 | Rural Recreation |  | X | Sport and Recreation |
| 7 | Advocacy |  | X | Transport and Human Movement Environment |
| 8 | Advocacy |  | X | Physical Environment, Urban Design and Liveability |
| 9 | Health Promotion Officer |  | X | Primary and Secondary Healthcare |
| 10 | Local Government Sport and Recreation Coordinator |  | X | Community-wide programmes |
| 11 | Health Promotion Officer |  | X | Primary and Secondary Healthcare |
| 12 | Local Tourism |  | X | Community-wide programmes |
| 13 | Health and Wellbeing Officer |  | X | Primary and Secondary Healthcare |
| 14 | Local Business |  | X | Workplaces |
| 15 | Secondary School Teacher |  | X | Education |
| 16 | Disability Sport and PA Officer |  | X | Sport and Recreation |
